# Pleistocene glacial cycle effects on the phylogeography of the Chinese endemic bat species, *Myotis davidii*

**DOI:** 10.1186/1471-2148-10-208

**Published:** 2010-07-10

**Authors:** Yuyan You, Keping Sun, Lijie Xu, Lei Wang, Tinglei Jiang, Sen Liu, Guanjun Lu, Sean W Berquist, Jiang Feng

**Affiliations:** 1 Key Laboratory for Wetland Ecology and Vegetation Restoration of National Environmental Protection, Northeast Normal University, Changchun, China; 2 Key Laboratory of Vegetation Ecology of Education Ministry, Institute of Grassland Science, Northeast Normal University, Changchun, China; 3 College of Urban and Environmental Science, Northeast Normal University, Changchun, China; 4 Department of Physiological Science, University of California, Los Angeles, USA

## Abstract

**Background:**

Global climatic oscillations, glaciation cycles and the unique geographic topology of China have profoundly influenced species population distributions. In most species, contemporary distributions of populations cannot be fully understood, except in a historical context. Complex patterns of Pleistocene glaciations, as well as other physiographic changes have influenced the distribution of bat species in China. Until this study, there had been no phylogeographical research on *Myotis davidii*, an endemic Chinese bat. We used a combination of nuclear and mitochondrial DNA markers to investigate genetic diversity, population structure, and the demographic history of *M. davidii*. In particular, we compared patterns of genetic variation to glacial oscillations, topography, and environmental variation during the Pleistocene in an effort to explain current distributions in light of these historical processes.

**Results:**

*M. davidii *comprises three lineages (MEP, SWP and SH) based on the results of molecular variance analysis (AMOVA) and phylogenetic analyses. The results of a STRUCTURE analysis reveal multi-hierarchical population structure in *M. davidii*. Nuclear and mitochondrial genetic markers reveal different levels of gene flow among populations. In the case of mtDNA, populations adhere to an isolation-by-distance model, whereas the individual assignment test reveals considerable gene flow between populations. MDIV analysis indicate that the split of the MEP and SWP/SH lineages, and from the SWP and SH lineages were at 201 ka BP and 158 ka BP, respectively. The results of a mismatch distribution analysis and neutrality tests indicate a population expansion event at 79.17 ka BP and 69.12 ka BP in MEP and SWP, respectively.

**Conclusions:**

The complex demographic history, discontinuous extant distribution of haplotypes, and multiple-hierarchy population structure of *M. davidii *appear associated with climatic oscillations, topography and eco-environmental variation of China. Additionally, the three regions are genetically differentiated from one another in the entire sample set. The degree of genetic differentiation, based on the analysis of mtDNA and nDNA, suggests a male-mediated gene flow among populations. Refuges were in the MEP, SH and the lower elevations of SWP regions. This study also provides insights for conservation management units (MEP, SWP and SH).

## Background

Global climatic oscillations and associated glaciation cycles have profoundly influenced species population distributions [1]. Pleistocene climate oscillations have contributed to the genetic structure of current species [2] and have played an important role in initiating intra-specific divergences of North American animal taxa [3]. However, climate condition in China during glacial periods appears to have been different from those of the rest of the world and bring about a series of eco-environmental effects, primarily due to the continuing uplift of the Qinghai-Tibet Plateau [4]. The uplift has heavily influenced the climatic and environment changes, and formed a complicated topography (a set of three ladders with the highest point to the west and the lowest to the east). The uplift has also had a decisive effect on the formation of the East-Asia monsoon, which increased the climatic differences between the glacial and interglacial period and influenced the extent of the glacial advance in China [4]. Additionally, climatic change induces changes in vegetation distribution, which also influences the distribution of animal populations through food chain and habitat modifications [5]. Thus, the distribution and change of forest vegetation may have influenced the migration, expansion and even extinction of animal populations which differed considerably from other regions of the world at the same latitude [4].

David's myotis (*Myotis davidii*) is endemic to China, occurring in a small province of middle and northern China [6]. This species is considered of least concern by IUCN [6]. The measurements of the bat specimen have on average 31.7 mm forearm length [7], 237.21 mm wingspan and weight 4.31 g (unpublished data). The wing morphology of this bat species is characterized by low wing loading and low aspect ratio, indicating that *M. davidii *has typically good manoeuvrability [8]. They prey on flying insects and inhabit rock or karst cavities [9]. Generally, cavities inhabited by this species are located in forests with high levels of insect diversity (unpublished results). However, there are no reports about migration, hibernation or ecological preferences. Previous research [10] on animal fossils of China has shown that the survival of bats depended mainly on forest cover. Furthermore, bat numbers or activity changes can be related to climate change [11]. Thus, bats are a good model for examining historical processes and distributional patterns following climatic oscillations.

In China, studies on the geographic distribution pattern of vertebrates, such as bats [12], birds [13] and amphibians [14], have described evolutionary histories following the global Pleistocene climatic oscillations and reflected differences with Europe in terms of evolution history in the context of complex regional scenarios [15]. Colonization events, dispersal patterns and migratory behaviours play a key role in determining a species' geographical distribution range and demographic history [15,16]. In addition, both historical events and ecological factors shape extant genetic diversity and population structure of animals [17]. Usually, analysis of genetic markers can be useful to successfully differentiate fine-scale structures in natural populations [16,18].

Different markers have their own unique genealogy, which may indicate concordance or divergence from the species' history. Mitochondrial DNA only reveals a species' maternal demographic history. However, microsatellite-derived data is influenced by sex-biased dispersal because of the character of biparental inheritance. Thus, the combination of different markers may actually provide complementary information to test the population demographic history [15] and extant population structure [18]. We therefore chose to include both nuclear DNA (nDNA) and mitochondrial DNA (mtDNA) analyses in our study of the phylogeography of *M. davidii*, for which genetic diversity, demographic history, and population structure remain unknown. Our main aims were to identify genetic diversity, describe macrogeographical population genetic patterns, and investigate the population demographic history within a context of climate oscillations, complex topology and eco-environmental variation since the mid-late Pleistocene. We inferred the historical causation of the extant population structure to better understand its distribution and population status. Additionally, we wanted to identify possible conservation management units and ultimately use information from the study to provide some advice on the further protection for *M. davidii*.

## Results

**Figure 1:**
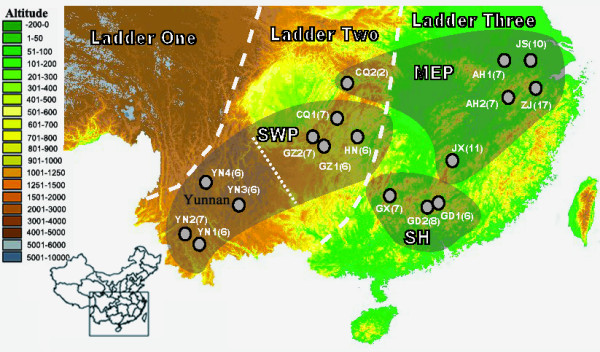
**Topographical map of China showing 17 sampling locations**. Samples sizes are shown on the map along with topographic boundaries and elevations.

Based on our research from 2001 to 2009, *M. davidii *was found to be scarce, but the distribution range was more extensive than previous records. Based on topography and physiography, populations of *M. davidii *can be divided into three regions: Middle East Plain (MEP), Southwest Plateau (SWP), and South Hills (SH) (Fig. 1 and Table 1).

**Table 1:** Genetic variability within the studied *Myotis davidii *populations based on mtDNA and nDNA data.

Clade	*h*	*π*	Province	Location	**Haplotype no**.	*n*	Coordinates	mtDNA				Microsatellite			
								
								*A*	*h*	*π*	**GenBank Accession No**.	*Ho*	*He*	*Rs*	*Fis*
MEP	0.994	0.041	Anhui	AH1	1-6	7	N31° 33' E118° 05'	6	0.950 ± 0.100	0.030 ± 0.019	GU013475-GU013480	0.50 ± 0.12	0.68 ± 0.13	1.43	0.25
			Anhui	AH2	7-12	7	N29° 47' E118° 10'	6	0.952 ± 0.096	0.022 ± 0.014	GU013481-GU013486	0.49 ± 0.22	0.63 ± 0.23	1.63	0.24
			Jiangsu	JS	3,13-14	10	N31° 22' E119° 48'	3	0.949 ± 0.044	0.050 ± 0.027	GU013477,GU013487,GU013488	0.59 ± 0.25	0.60 ± 0.23	1.52	0.05
			Zhejiang	ZJ	14-26	17	N30° 06' E120° 02'	13	0.600 ± 0.131	0.028 ± 0.016	GU013488,GU013489-GU013500	0.54 ± 0.37	0.69 ± 0.17	1.43	0.04
			Jiangxi	JX	27-34	11	N26° 36' E114° 12'	8	0.956 ± 0.054	0.027 ± 0.015	GU013501-GU013508	0.52 ± 0.11	0.71 ± 0.17	1.44	0.13
			Chongqing	CQ2	35	2	N30° 58' E108° 08'	1	-	-	GU013509	0.53 ± 0.01	0.56 ± 0.03	1.50	0.11

SWP	0.972	0.023	Chongqing	CQ1	36	7	N29° 16' E107° 50'	1	-	-	GU013510	0.43 ± 0.26	0.58 ± 0.18	1.51	0.17
			Hunan	HN	37-41	6	N28° 17' E109° 39'	5	0.810 ± 0.172	0.015 ± 0.009	GU013511-GU013515	0.58 ± 0.22	0.71 ± 0.29	1.62	0.19
			Guizhou	GZ1	42-45	6	N27° 59' E107° 11'	4	0.600 ± 0.175	0.007 ± 0.005	GU013516-GU013519	0.70 ± 0.42	0.74 ± 0.10	1.47	0.05
			Guizhou	GZ2	46-47	7	N28° 23' E106° 25'	2	0.997 ± 0.127	0.032 ± 0.020	GU013520,GU013521	0. 63 ± 0.22	0.73 ± 0.24	1.73	0.18
			Yunnan	YN1	1,13	6	N22° 36' E100° 43'	2	0.500 ± 0.250	0.004 ± 0.003	GU013475,GU013487	0.69 ± 0.34	0.75 ± 0.13	1.75	0.02
			Yunnan	YN2	1	7	N22° 46' E100° 05'	1	-	-	GU013475	0.57 ± 0.29	0.72 ± 0.20	1.63	0.22
			Yunnan	YN3	1	6	N25° 03' E102° 42'	1	-	-	GU013475	0.55 ± 0.26	0.71 ± 0.12	1.54	0.25
			Yunnan	YN4	13	6	N26° 28' E100° 50'	1	-	-	GU013487	0.61 ± 0.32	0.70 ± 0.19	1.60	0.22

SH	0.951	0.022	Guangxi	GX	48-49	7	N25° 24' E110° 41'	2	0.730 ± 0.220	0.007 ± 0.004	GU013522, GU013523	0.61 ± 0.36	0.69 ± 0.29	1.60	0.13
			Guangdong	GD1	50-51	6	N24° 46' E113° 34'	2	0.930 ± 0.165	0.012 ± 0.008	GU013524, GU013525	0.57 ± 0.19	0.59 ± 0.26	1.53	0.24
			Guangdong	GD2	50,52-53	8	N24° 44' E113° 31'	3	0.453 ± 0.245	0.025 ± 0.019	GU013524, GU013526, GU013527	0.44 ± 0.21	0.52 ± 0.27	1.49	0.23

MEP represents Middle East Plain; SWP represents Southwest Plateau; and SH represents South Hills. The number of individuals per population (*n*), number of haplotypes (*A*), haplotype diversity (*h*), nucleotide diversity (*π*), observed heterozygosities (*H*_O_), expected heterozygosities (*H*_E_), allelic richness (*R*_S_), inbreeding coefficients (*F*_is_).

### mtDNA and nDNA genetic diversity

Examination of mitochondrial HVI sequences showed length variation (579-909 bp). The R1 repeat (consisting of three to seven 81 bp repeats) within mtDNA HVI region which responsible for the length variation of HVI region, were excluded from the analysis. After exclusion of the tandem repeats, the 340 bp sequence of the HVI region revealed 53 different haplotypes, defined by variation at 159 polymorphic sites. Thirty-eight of these polymorphisms were the result of indels, and 72 polymorphic sites were parsimony-informative. Haplotype diversity (*h*) averaged over all populations 0.975 ± 0.002. Haplotype diversity (*h*) was uniformly high and was the highest in the MEP region (Table 1). Most haplotypes were unique (36 haplotypes, 28.57% of the individuals). Other haplotypes were shared within populations (17 haplotypes, 69.84% of the individuals), among populations (5 haplotypes, 38.89% of the individuals) or among regions (2 haplotypes, 21.43% of the individuals; AH1 shared with YN1/YN2/YN3 and JS shared with YN1/YN4) (Additional file 1). Nucleotide diversity (*π*) averaged over all populations 0.062 ± 0.007. Patterns of variation in nucleotide diversity across regions were consistent with that of haplotype diversity in that the greatest diversity was found in the MEP and the lowest in the SWP and the SH lineages (Table 1).

Analysis of nDNA genetic diversity indicated that observed heterozygosity ranged from 0.43 to 0.70, and allelic richness ranged from 1.43 to 1.75 among populations (Table 1). We did not detect any statistically significant deviations from HW equilibrium among the eight loci (Table 2). No genotypic linkage disequilibrium was found between or within population samples. None of the inbreeding coefficients (*F*is) were significant at the population level (Table 1), suggesting random mating within populations and the absence of null alleles.

**Table 2:** Characteristics of eight microsatellite loci for *Myotis davidii*.

Locus	Primer[μM]	MgCl_2_[μM]	Primer sequence (5'-3')	Fragment size (bp)	HW *P *value	Fluorescein tag
A13	0.45	2	F: AACGTTCATTCTGCCAAAGG	409-421	0.849	FAM
			R: TCATGCTGTTCCACTTCTGG			
E24	0.25	1.5	F: GCAGGTTCAATCCCTGACC	220-228	0.841	FAM
			R: AAAGCCAGACTCCAAATTCTG			
H29	0.35	1.5	F: TCAGGTGAGGATTGAAAACAC	164-188	0.95	FAM
			R: GCTTTATTTAGCATTGGAGAGC			
G9	0.25	1.5	F: AGGGGACATACAAGAATCAACC	162-178	0.9912	FAM
			R: TAATTTCTCCACTGAACTCCCC			
D9	0.25	2	F: TCTTTCCTCCCCTGTGCTC	104-136	0.812	HEX
			R: TCTGGACCCAAAATGCAGG			
G25	0.25	1.5	F: TCCTTCCCATTTCTGTGAGG	131-137	0.861	HEX
			R: CCATTTCATCCATCCAGTCC			
G30	0.25	1.5	F: TTGCCAAATTCTGGTATCTTCC	130-156	0.6029	HEX
			R: AGAGCTTAATGGGGAGGCTG			
C113	0.25	1.5	F: ACCTCCCTGCCCTGCAC	98-102	0.74	HEX
			R: GCAATGCTTCCTCCAAGTCC			

### Phylogenetic and demographic analysis

**Figure 2:**
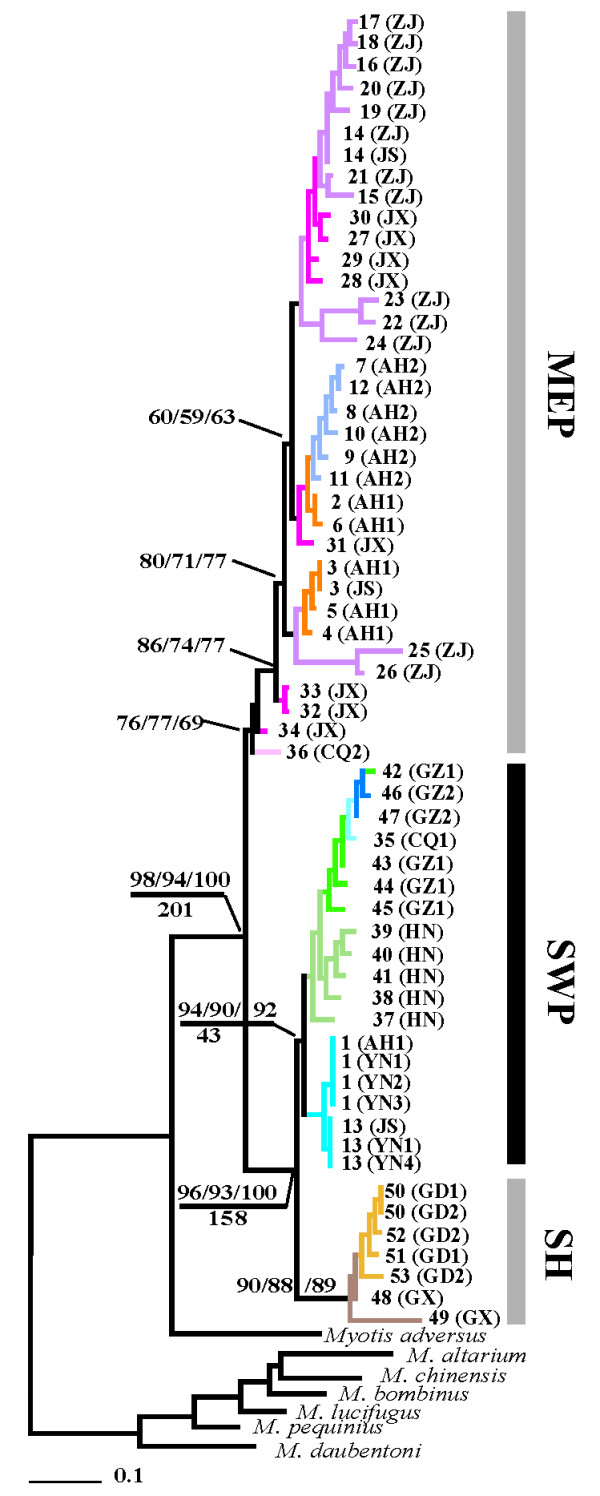
**Maximum-likelihood phylogenetic tree of *Myotis davidii***. Maximum parsimony (MP), maximum likelihood (ML) and Bayesian inference (BI) of our dataset resulted in concordant topologies (bootstrap values are above the line and divergence times (ka BP), estimated from MDIV, are below the line). Only Bootstrap values above 50% are shown.

Maximum parsimony (MP), maximum likelihood (ML) and Bayesian inference (BI) yielded highly concordant results (Fig. 2). Each tree revealed that *M. davidii *formed a monophyletic lineage with respect to congeneric species (*M. daubentoni*, *M. lucifugus*, *M. adversus*, *M. altarium*, *M. bombinus*, *M. chinensis*, *M. pequinius*). In three trees, haplotypes grouped into two clades, representing the SWP/SH and MEP lineages (Fig. 2). Divergence time was estimated to be 201 ka (95% credibility interval 280-113 ka BP) based on the results of MDIV analysis. The former lineage was further subdivided into two subclades, SWP and SH (Fig. 2), for which the divergence time was estimated to be 158 ka BP (95% credibility interval 230-77 ka BP).

In regard to the neutrality tests, negative values of Tajima's *D *and Fu's *Fs *in MEP (*P*_D _= 0.005, *P*_Fs _= 0.026) and SWP (*P*_D _= 0.048, *P*_Fs _= 0.049) were all significantly deviated from "0" (Table 3), which can be interpreted as a signal of demographic expansion in both regions. However, SH and the populations in the whole distribution range did not indicate demographic expansion based on the non-significant *P *values of Tajima's *D *and Fu's *F*s (Table 3). A mismatch distribution analysis also indicated population expansion events in MEP and SWP based on a small and nonsignificant *SSD *and *r *values (Table 3). The population expansion time was 79.17 ka BP and 69.12 ka BP in MEP and SWP, respectively (Table 3).

**Table 3:** Demographic history analysis of *Myotis davidii*

	Neutrality tests				Mismatch distribution analysis				Tau (CI = 95%)	*t *(ka BP)
			
	Tajima's *D*	*P*_D_	Fu's *F*s	*P*_Fs_	SSD	*P*_SSD_	*r*	*P*r		
MEP	-2.103	0.005	-8.855	0.026	0.010	0.329	0.010	0.277	10.77 (4.264-23.822)	79.17 (31.354-175.167)
SWP	-0.591	0.048	-21.980	0.049	0.025	0.156	0.058	0.075	9.40 (4.771-13.080)	69.12 (35.082-96.179)
SH	-1.410	0.084	-11.077	0.203	0.456	0.038	0.140	0.232	-	-
Total	-1.361	0.131	-3.856	0.206	0.030	0.262	0.069	0.192	-	-

### Population structure

An AMOVA revealed significant genetic variance for all three hierarchical levels examined (among regions, among populations within regions, and within populations) (Additional file 2). The grouping pattern was MEP (composed of AH1, AH2, JS, ZJ, JX, CQ2), SWP (composed of CQ1, GZ1, GZ2, HN, YN1, YN2, YN3, YN4), and SH (composed of GD1, GD2, GX) gave the highest Φ_CT _value (0.771, *P *< 0.01). Most of the genetic variance (64.82%) was explained by differences among the three regions (Additional file 2). The subdivisions depicted by the AMOVA are consistent with the macrogeographical structure of China (Fig. 1). In addition, the analysis of AMOVA highlighted a low but significant microsatellite genetic variation (14.45%) among the three regions and a high proportion of genetic of variation (68.98%) within populations (Additional file 2). In mtDNA, the pairwise genetic differences among populations varied from 0.007 to 0.988 (*P *< 0.01) (Additional file 3), and only 3.13% of pairwise genetic differences were not significant in the whole population. In nDNA, the pairwise genetic difference among populations varied from 0.004 to 0.276 (*P *< 0.01) (Additional file 3), and 82.14% pairwise genetic differences in the SWP lineage were not significant. Thus the pairwise genetic differences among populations within regions were smaller than among regions in both markers (Additional file 3). These results suggest that the genetic differentiation maximizes among the three regions.

A Mantel test indicated that genetic subdivision within *M. davidii *fits an isolation-by-distance model (*R*^2 ^= 0.296, *P *= 0.031) with respect to mtDNA. However, nuclear markers failed to support isolation-by-distance model (*R*^2 ^= 0.030, *P *= 0.895).

**Figure 3:**
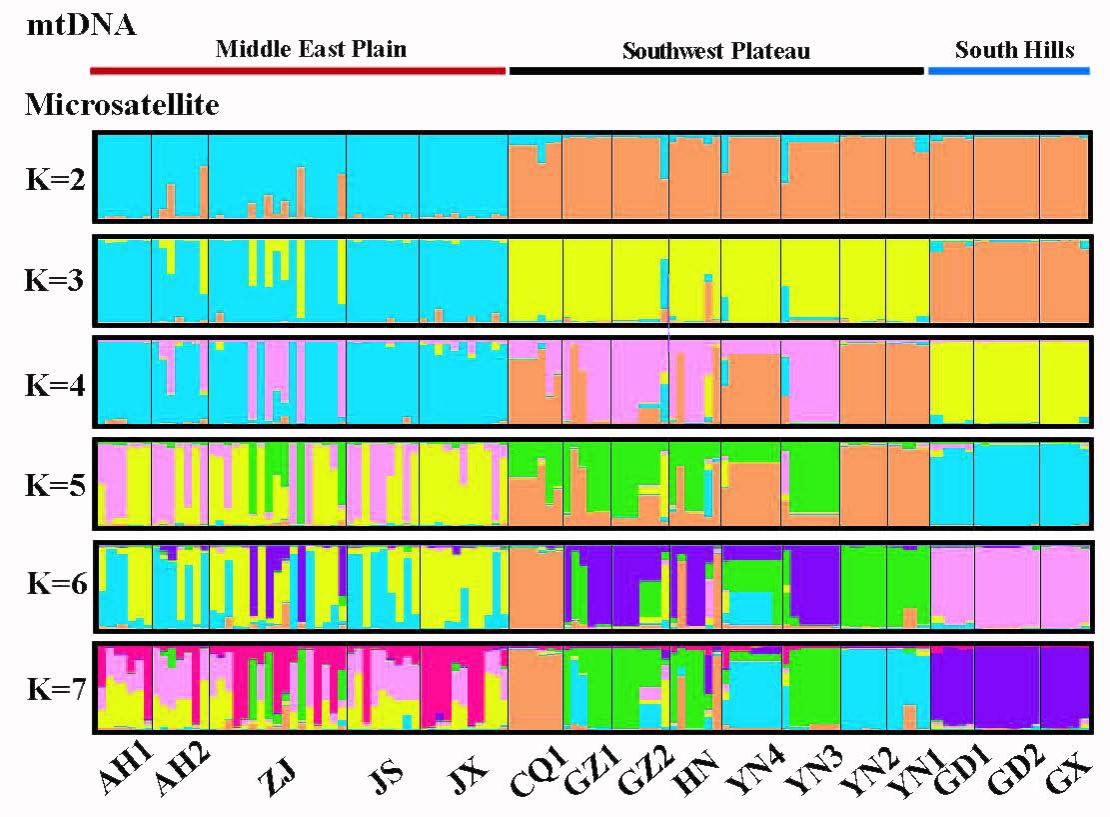
**Graphic derived from the program STRUCTURE**. We repeated the Bayesian clustering analysis with STRUCTURE and assigned individuals into clusters at values of *K *beyond the number considered to maximize the posterior probability and reconstruct the hierarchical relationship among populations. Each individual is represented by a vertical line which is partitioned into *K *colored segments, the length of each color being proportional to the estimated membership coefficient. Black lines separate individuals of different populations as indicated by the labels at the bottom of the figure. Graphical represented clusters for samples in the Middle East Plain, Southwest Plateau and South Hills. Each individual is depicted by a horizontal line, which is partitioned into *K *colored sections. Labels above the figure indicate the three lineages (MEP, SWP and SH) based on ML tree.

Due to its small sample size, the CQ2 population (n = 2) was excluded from the STRUCTURE analysis. Clustering of individuals based on their multi-locus genotypes revealed substantial hierarchical structure among populations across the species range (Fig. 3). When we assigned individuals to clusters at *K *= 2 clustering recovered two groups, which is in agreement with the ML tree lineages (MEP *vs*. SWP/SH) (Fig. 2 and Fig. 3). At *K *= 3, the SWP and SH groups formed a distinct cluster which is in agreement with the sub-lineages of ML tree (SWP *vs*. SH). Individuals from SH region showed the least similarity with the MEP region. SWP region was observed to be separated into two clusters at *K *= 4, represented by a relatively low-elevation plateau (YN1-YN2) and a relatively high-elevation plateau, with evidence of a cline in membership between these clusters. An additional division was detected between CQ1 and other populations in the SWP region at *K *= 6. The separation at *K *= 4 and *K *= 6 was not the same as the ML tree lineages. The division was detected between AH2-ZJ and other populations in the MEP group at *K *= 2, however, a marked separation was found between AH2 and ZJ in the ML tree. Within the MEP region, individuals were assigned to two or more populations at *K *= 5-6, whereas they can trace ancestry to more than one population. The results of individual assignment test indicated a frequency gene flow within the MEP region. No marked variation in the population structure was detected at *K *≥ 7.

Individual assignment test indicated that a greater proportion of individuals of *M. davidii *(63.49%) were assigned to the populations from which they were sampled. Within the MEP, SWP and SH regions, 44.44%, 23.53% and 19.05% of the individuals, respectively, were assigned to other populations. Thus, results of the individual assignment test also pointed toward higher gene flow among populations within regions, especially within the MEP region. When using individual assignment tests according to the three mtDNA clades, one individual (0.79%) in the SWP (HN) group were assigned to the SH (GD2) group. Two individuals (1.58%) in the MEP (ZJ) group were assigned to the SWP (YN3/YN4) group. Three individuals (2.38%) in the SWP group were assigned to the MEP group, where YN3 and GZ2 originated from ZJ/JS and JX, respectively. Thus, 4.75% of individuals were assigned to other lineages, which indicated a weak gene flow among the three regions.

## Discussion

### Genetic structure of populations

Compared with similar study on bat mtDNA [19], *M. davidii *showed higher mean nucleotide diversity (mean π = 0.062 ± 0.007). The presence of high *h *and high π indicated that a sub-lineage formed over long periods of evolutionary time [20]. In addition, a high *h *and high *π *indicated high levels of divergence within three regions, which was attributed to secondary contact between previously differentiated allopatric lineages. Like other bat species [18,19], *M. davidii *was not detected the continuity gene flow among populations based on mtDNA. In addition, isolation by distance was an important factor for the formation of genetic structure within maternal populations. The pattern of subdivision into subpopulations can be explained by the influence of environmental characteristics. In China, the west-east axis was a direction of the prominent changes in many environmental variables, such as vegetation types, temperature, precipitation, and topography, all of which were due to the continuing uplift of the Qinghai-Tibet Plateau. The dependence of genetic differentiation on a gradient of environmental variables is in agreement with the theoretical model of Doebeli & Dieckmann [21], showing that processes of evolutionary diversification may lead to sharp geographical differentiation along environmental gradients. The environmental gradients existed for an extended period of time which reflected in mtDNA.

However, the population genetic structure in microsatellite loci was less pronounced than in the case of mtDNA analysis. Bayesian clustering analysis (Fig. 3) and individual assignment test showed frequent migration within regions. And the patterns of migration followed a stepping-stone colonization model. Large and non-significant *F*_is _values also showed frequent transfer in males among populations and led to an unobvious population structure. Patterns of genetic differentiation in two molecular markers were attributed to male dispersal and female philopatry [22,23]. Male-biased gene flow implied low introgression of mtDNA haplotypes from neighboring populations, and therefore greater structuring in mtDNA as compared with nuclear markers.

Our comparisons showed that the deepest phylogenetic split (SWP/SH vs. MEP, Fig. 2) among the haplotypes corresponded exactly to nDNA-based structure when genotypes were in two groups (*K *= 2, Fig. 3). The deepest phylogenetic split was similar to the pattern obtained from *Rhinolophus ferrumequinum *in China [15]. SWP and SH lineages split during stage III of the glacial epoch in the Yun-Gui plateau (154-136 ka BP) [24], and separated into two geographically defined lineages (Fig. 2), which was in agreement with the results of the nDNA analysis (*K *= 3, Fig. 3). The population genetic structure was consistent with topographic and geographic characteristics of China. Thus, ecological environmental changes, landscape structure differentiation, and the climate discrepancy are primary determinants of population boundaries and rate of movement among regions [25,26].

When these regions were considered separately we found clear differences in the phylogeographical signal obtained from the two sets of markers. Multi-locus genotyping data suggest that *M. davidii *has multiple levels of population structure (Fig. 3), which were not shown by mtDNA. Compared with mtDNA, the concordance between the two markers seemed to break down within the SWP clade. These similarities and discrepancies between the data sets together clarify the history of this species within the SWP region. In the SWP, four sampling locations in Yunnan, formed an independent lineage (Fig. 2), which coincided with the elevation difference of other populations (GZ, CQ and HN; Fig. 1). This result is in agreement with previous studies on birds [13] and amphibians [14]. However, nDNA analysis indicated that the separation of the YN1-YN2 *vs*. other populations in the SWP group was maintained following the introduction of a fourth and fifth microsatellite-based cluster, which indicated much more frequent contact between YN1 and YN2 populations than between these two and any other population. Additional discordance at *K *= 4 indicated postglacial colonization across the whole range of SWP region with evidence of a cline in membership between these clusters. Discordance at *K *= 6 indicated that CQ1 population might be isolated by geographical barrier, although CQ1 and other locations (HN, GZ1 and GZ2) were all in the same lineage of the phylogenetic tree (Fig. 3). These clusters in YN1-YN2 and CQ1 may represent a discrete, discontinuous jump across a relatively small geographical distance. The gene exchange among *M. davidii *populations in SWP also may be impeded by the limit of landscape.

In the MEP region, no obvious population structure was found at the higher level of *K *= 5-7, which indicated a continual population contact with one another. Extensive and overlapping ranges within the MEP region of the Bayesian clustering analysis (Fig. 3) indicated the absence of geographical features in the landscape that would not constitute efficient barriers for bat dispersal. SH also showed no clear population division and indicated a much more stable population structure than that of MEP.

### Population demographic reconstructions

Since the Middle and Late Pleistocene, violent glacial-interglacial cycles (at least 24 Dansgaard-Oeschger cycles and several Heinrich events between 115 ka BP and 14 ka BP; on average, each fluctuating glacial cycles persisted for about 1500 years [27]), complicated topology and eco-environmental changes due to the Qinghai-Tibet Plateau uplift [4] had important influences on the demographic history of many species [12-15]. With the complexity climatic conditions due to glacial oscillation and Qinghai-Tibet Plateau uplift, Chinese species have experienced a unique evolutionary history [4].

Based on the divergence time estimated among mtDNA lineages, the demographic history of *M. davidii *could be traced back to before the Riss glaciation (210-135 ka BP) during the Pleistocene [28], which was similar to the glacial epoch in middle, southern and southwestern China (223-189 ka BP) [29]. Both markers thus support the scenario that the SWP and SH groups have a similar history and a common origin, while a parallel evolving history is observed for the SWP/SH and MEP lineages.

In the MEP lineage, a population expansion event of *M. davidii *was occured around 79.17 ka BP (Table 3). This expansion thus took place during the end of the last interglacial period (130-75 ka BP), one of the warmer periods [29]. The expansion time was younger than of the greater horseshoe bat (*Rhinolophus ferrumequinum*) for Japan (127-191 ka BP) [15] and was nearly the same as amphibians of the same region (80 ka BP) [14]. The different expansion time with the greater horseshoe bat may be due to the influence of glacial oscillations at different latitudes at the same time [30]. Bats are sensitive to environmental changes [11], such as the large-scale marine transgressions in the MEP region [31], which may influence bats' migratory behaviour and geographic distribution [32]. Thus, a stepwise population expansion, inferred by individual assignment test of nDNA, has taken place in association with eco-environmental changes.

In the SWP region, a population expansion event of *M. davidii *occurred around 69.12 ka BP (Table 3), and originated from relatively high elevation areas in SWP to the MEP during 72-60 ka BP (early stage of the last glacial) [33]. The expansion time was a little earlier than that of the greater horseshoe bat (*R. ferrumequinum*) for Europe (40-60 ka BP) and during the same period for Russia (14-81 ka BP) [15]. Two haplotypes (21.43% individuals) were shared between YN populations in the SWP and AH1-JS populations in the MEP region. This conclusion is in agreement with previous studies on bats [12], birds [13] and amphibians [14]. The result of nDNA analysis (Fig. 3) and an individual assignment test also indicated that some individuals of *M. davidii *shared the same nDNA alleles in the SWP and MEP regions, and no cline across these populations. The high elevation areas of the SWP region were the first to be affected by the sudden cold weather caused by the lowering of the snow line during the last glacial period (74-11.5 ka BP) [34]. Therefore, some species or individuals in high altitudes may have immigrated to refuges, such as Yunnan, or to other regions in order to avoid such cold climatic conditions.

Based on the analysis of microsatellite data, a possible regional population contact was interpreted, which showed a similar allele site among populations in the SH group (Fig. 3). Although the phylogenetic tree was interrupted between SH and SWP, the relationship between SWP and SH groups was much closer than MEP based on the mtDNA results (Fig. 2), which may be an indication of SWP and SH origin from a common ancestor.

### Refuges for *Myotis davidii*

At least two refuges have been reported in east China and in the lower elevations of the southwestern plateau [14,35]. In our study, the existence of three mitochondrial lineages, with high nucleotide and allelic diversity directed towards three relict refuges for *M. davidii *in China, where an initial population expansion took place. There is no clear indication of where the refuge of *M. davidii *populations were located, but the possible areas are the low-elevation plateau, such as Yunnan area and Sichuan Basin (CQ2 is at its margin), where there were large-scale relict refuges for many species [36]. In our study, the results of nDNA analysis imply a relatively simpler genetic structure for *M. davidii *in YN1-YN2 and CQ1, which is attributed to relict refuge during the last glaciations. The eastern coast and south hills all were glacial refuges which were indicated by the high nucleotide and allelic diversity of both markers. This trend also has been demonstrated for other species [11].

### Protection considerations

Genetically divergent populations are increasingly being recognized as appropriate units for conservation. The results presented here potentially indicate three conservation management units (MEP, SWP and SH). For the different management units, we suggest that protection require several different measures. As for the MEP region, in considering frequent contact among populations, there may be no barrier in population migration due to the lack of mountains. Therefore, it is important to strengthen the protection of these habitats. In the SWP and SH regions, the gene exchange among *M. davidii *populations may be impeded by the limit of landscape. Therefore, it is important to protect migration corridors. In addition, CQ2 is located in the Three Gorges Area at the entrance of the Sichuan basin. Small mammal fossils in the Three Gorges Area indicate that it provids a corridor for the exchange of fauna occurring in temperate and more tropical areas to the south [10]. The position of CQ2 in the phylogenetic tree also points to a corridor of genetic exchange between the MEP and SWP/SH regions. In addition, the finding that *F*_st _and Φ_st _values between CQ2 and other populations within the MEP region exceed values among all the other populations suggests that populations of *M. davidii *are especially vulnerable to small population effects and fragmentation. A corridor of genetic exchange, such as CQ2, and prey or colony habitats should be protected to maintain population contact and its population persistence.

## Conclusions

*M. davidii *is considered to be of least concern by IUCN [6]. Also, before 2009 there were no systematic studies made of the distribution limit, status, and phylogeography of Chinese endemic bat species. Our research has shown that the distribution range of this species is more extensive than indicated by previous records. *M. davidii *also were found to be scarce and were not found in most recorded locations during the last nine years. Therefore, the public, environmental protection agencies, and various stakeholders should spark efforts for its future protection.

High haplotype and nucleotide diversity indicate that *M. davidii *has had a long evolutionary history. Based on the analysis of MDIV, the differentiation times among the three regions were during the Riss glaciation (210-135 ka BP), and the population differentiations correspond to a series of geological events and glacial cycles. The populations in the MEP and SWP experienced expansion events at about 79.17 ka BP and 69.12 ka BP, respectively. The phylogeographic study showed deep genetic differentiations among populations of *M. davidii*. Glacial cycles, ecological barriers and topological differences are associated with the extant distribution patterns and phylogenetic clades of *M. davidii*. The populations of *M. davidii *formed multihierarchical population structures and also conserved high genetic diversity in each region. The degree of genetic differentiation, based on the analysis of mtDNA and nDNA of *M. davidii*, suggests a male-mediated gene flow among populations or regions.

## Methods

### Sampling and Mitochondrial DNA amplification

We initiated our investigation of *M. davidii *in 2001. For 9 years, 126 individuals of *M. davidii *were collected from 17 localities (Fig. 1). All tissues were collected with a non-lethal method: this involved taking a wing punch biopsy for DNA analysis [37]. Protocols for capturing and handling live bats followed the Regulations of Wildlife Conservation of the People's Republic of China (No. 24) [38], which were approved by the Wildlife Conservation Association of China.

Total genomic DNA was isolated from tissue using the Qiagen DNAeasy Tissue Kit. Hypervariable region I (HVI) of mitochondrial control region was amplified by polymerase chain reaction using primer pair P-F [39]. In order to guarantee accurate sequencing, all amplification and sequencing was repeated at least once. All PCR reactions were performed in 25.0 μL volumes, containing approximately 10-30 ng of genomic DNA, 2.5 mM MgCl_2_, 75 mM Tris-HCl (pH9.0), 20 mM (NH_4_)_2_SO_4_, 0.01% (v/v) Tween-20, 0.2 mM dNTP mix, 10 μM of each primer and 1U of Bioline Taq polymerase. The following reaction conditions were used: 3 min at 94°C; 40 cycles of 94°C for 1 min, 55°C for 1 min, 72°C for 1 min; and 72°C for 10 min. DNA sequencing was performed on an ABI 3730 automated DNA sequencers (Applied Biosystems). DNA sequences were edited and aligned using BioEdit 7.0.5.3 [40]. All haplotypes were submitted to GenBank [GenBank: GU013475-GU013527].

### Microsatellite genotyping

Subsequent to screening 16 microsatellite loci originally isolated from either *Myotis myotis *[41] or *M. daubentonii *[42], 8 polymorphic loci were selected for examining genetic variation in *M. davidii *(Table 2). All forward primers were labelled with 5'-Fluorescent bases (Table 2), and PCR reactions were conducted in 10 μL volumes containing 10 ng of template DNA, 1.5 mM MgCl_2_, 75 mM Tris-HCl (pH 9.0), 20 mM (NH_4_)_2_SO_4_, 0.01% (v/v) Tween-20, 0.2 mM dNTP mix, 1 μM of each primer and 0.5 U of Bioline *Taq *polymerase. Touchdown PCR was used with all primers, with an initial denaturing step of 3 min at 94°C. The annealing temperature of the reaction is decreased 0.5°C each cycle from 60°C to a touchdown at 50°C, at which temperature 20 cycles are carried out. An additional 25 cycles were then performed with 30 s denaturing at 91°C and 30 s annealing at 50°C. No extension steps were included [43], apart from a 5 min period at 72°C following the final annealing step. Genotyping was performed on an ABI 3730 automated DNA sequencer (Applied Biosystems, Inc.).

### Genetic diversity

Estimates of mitochondrial haplotype variability within different regions and populations included the number of haplotypes (*A*), haplotype diversity (*h*) and nucleotide diversity (*π*). All estimates were made using ARLEQUIN 3.1 [44]. Values for polymorphic sites and parsimony informative sites were estimated in DnaSP, version 4.0 [45].

Microsatellite genotype data were scored using the Genemarker software 1.75 (SoftGenetics Inc.). Genetic diversity was assessed for each population by calculating expected heterozygosity (*H*_E_), observed heterozygosity (*H*_O_) and average allelic richness (*R*_S_) determined with FSTAT 2.9.3 [46]. Multi-locus *F*_is _was calculated for each population and adjusted for multiple tests using a Bonferroni's correction [47]. Deviation from the Hardy-Weinberg equilibrium (HWE) was tested by permutation using FSTAT 2.9.3 [46]. Tests for linkage disequilibrium between loci for each population were performed with GENEPOP 3.4 [47].

### Phylogenetic analysis

Phylogenetic analyses of unique haplotypes included both maximum parsimony (MP) and maximum likelihood (ML) algorithms performed in PAUP 4.0b10 [48]. A GTR + G + I model was used, as determined by Modeltest 3.7 [49] (base frequencies: A, 0.3870; C, 0.2174; G, 0.1122; and T, 0.2834; transition/transversion ratio = 4.9960; proportion of invariable sites Pinvar = 0.0892; gamma distribution shape parameter = 2.4244). MP analyses were conducted using a heuristic search option with 100 random sequence additions and tree-bisection-reconnection (TBR) branch swapping. Robustness of the MP trees was assessed by 1000 bootstrap replicates. ML analyses used parameters estimated from trees obtained from MP analyses. ML analyses used heuristic searches with starting trees obtained by NJ followed by TBR branch-swapping. ML nonparametric bootstrap analyses used 100 heuristic searches with starting trees obtained with NJ based on *p *distances followed by TBR and nearest-neighbor interchange branch-swapping, saving all optimal trees. Bayesian inference was performed with MrBayes 3.1 [50] using default parameters. Two independent parallel runs of four incrementally heated Metropolis-coupled MCMCs (Monte Carlo Markov Chains) were performed, with trees sampled every 100 generations for 1000000 generations to the average standard deviation of split frequency below 0.01. The first 10% of the trees were discarded as 'burnin', and posterior probabilities were estimated for the remaining saved trees. Trees were rooted with sequences of *M. daubentoni*, *M. lucifugus*, *M. adversus*, *M. altarium*, *M. bombinus*, *M. chinensis *and *M. pequinius *[GenBank: EU447268, U95342, U95341, U944765, GU944764, GU936479, GU944763].

### Demographic history

The population demographic expansion was tested by mismatch distribution analysis and neutrality tests. Mismatch distribution analyses were implemented to detect historical demographic expansions. Significant difference from a model of sudden expansion was assessed using the sum of squared deviations (*SSD*) and the Harpending raggedness index (*r*) with parametric bootstrapping (10000 replicates). Generally, a smaller and non-significant value (*P*_SSD _and *P*_r _> 0.05) indicated population expansion. We estimated the time since expansion (*t*) with the equation *τ *= 2 *μkt *[51], where *τ *(tau) is a parameter of the time to expansion in units of mutations, *μ *is the mutation rate per nucleotide and *k *is the number of nucleotides of the sequence. Tajima's *D *and Fu's *F*s (based on the infinite-site model) were sensitive to the population expansion. Significant negative values of Tajima's *D *were interpreted as non-neutral conditions. Negative values of Tajima's *D *and Fu' *F*s significantly deviated from "0", inferred as a signal of demographic expansion. All analyses were conducted with ARLEQUIN 3.1 [44].

With a coalescent-based approach, the program MDIV was used to estimate the timing of population divergence [52]. MDIV was implemented using a 'finite model' (HKY), with 5000000 Markov chain iterations and a 25% burn-in. Mmax and Tmax were set to 10 and 5, respectively. The divergent times (*t*) were estimated using the formula *t *= T_pop_*(*θ*/2 *μk*), where *T_pop _*is the time of population divergence, *θ *is the population mutation rate, *μ *is mutation rate per nucleotide, and *k *is the number of nucleotides of the sequence. Likelihood values for *T_pop _*and *θ *were calculated and the value with the highest posterior probability accepted as the best estimate [14]. The program was run multiple times with different random seeds to obtain consistent distribution results.

We used a mutation rate of 20% per million years (Myr) in our study, which was previously applied to bats of the genus *Nyctalus *[53]. The generation time was estimated to be 2 years.

### Population structure analysis

Genetic differentiation among populations was quantified by computing pairwise differentiation Φ_st _for mtDNA using ARLEQUIN 3.1 [44]. *F*_st _was calculated with GENALEX 6.0 for microsatellites [54].

To test for geographical genetic structure, analyses of molecular variance (AMOVA) with 10000 permutations were assessed according to the degree of differentiation between regions (Φ_CT_), between populations within regions (Φ_SC_) and between all populations (Φ_ST_) by using ARLEQUIN 3.1 [44].

To compare patterns of structure obtained from mtDNA haplotype data with those from multi-locus markers, we used STRUCTURE 2.2 [55] to reconstruct the hierarchical relationship among populations, as well as to distinguish historical processes. We applied a mixed model that allows for allele frequency correlation across a set of *K *genetic clusters. We performed 10 replicate [15] runs of structure for each value of *K*. We applied the admixture model with a burn-in of 30000 and a run length of 10^6^. Summary outputs for each value of *K *were then displayed graphically using DISTRUCT [56]. Assignment tests can be used to identify the source population of individuals, and individuals of unknown origin can be pre-assigned to populations according log likelihood scores [57]. Therefore, we used assignment tests to identify the origin population according to likelihoods from genotypic data [57], which was implemented with GENALEX 6.0 [54].

A Mantel test (10000 permutations) to assess the statistical significance of the correlation between genetic and geographical distances was performed with IBDWS 3.14 [58], to understand the influence of geographical barriers on population differentiation.

## Authors' contributions

YY carried out the molecular genetic analysis, participated in manuscript's design and drafted the manuscript. KS, LX, LW, TJ, SL, GL, JF participated in the design of the study and performed the statistical analysis. Sean W. Berquist participated in manuscript editing. All authors read and approved the final manuscript.

## Supplementary Material

Additional file 1 **mtDNA haplotypes**. Distribution of dloop region haplotypes shared in the same and different local populations of *Myotis davidii*. Click here for file

Additional file 2 **AMOVA in mtDNA and microsatellite**. The molecular variance analysis (AMOVA) in mtDNA and microsatellite of *Myotis davidii *were in three geographical regions. Regions chosen: Middle East Plain, Southwest Plateau, South Hills. Asterisks highlight hierarchical levels explain a significant proportion of the overall variance (*P *< 0.001). Click here for file

Additional file 3 **Estimates of genetic difference derived from both mtDNA and microsatellites**. Above diagonal is Φ_st _from mtDNA. Below diagonal is *F*_st _from microsatellites. The significant values are marked with asterisks. The pairwise genetic difference within regions is in bold. Significance level = 0.05. Click here for file
