# Supplementary material for: Pleistocene glacial cycle effects on the phylogeography of the Chinese endemic bat species, Myotis davidii
Source: BMC Evol Biol. 2010 Jul 10;10:208. doi: 10.1186/1471-2148-10-208 (PMC3055248; doi:10.1186/1471-2148-10-208)
Supplement: Additional file 1 — mtDNA haplotypes. Distribution of dloop region haplotypes shared in the same and different local populations of Myotis davidii. [file 1471-2148-10-208-S1.DOC]

**Additional file 1**

***mtDNA haplotypes.*** *Distribution of dloop region haplotypes shared in the same and different local populations of Myotis davidii.*

| Hap No. | Middle East Plain | | | | | | Southwest Plateau | | | | | | | | South Hills | | |
| --- | --- | --- | --- | --- | --- | --- | --- | --- | --- | --- | --- | --- | --- | --- | --- | --- | --- |
|  | AH1 | AH2 | JS | ZJ | JX | CQ2 | CQ1 | YN1 | YN2 | YN3 | YN4 | GZ1 | GZ2 | HN | GX | GD1 | GD2 |
| 1 | 1 |  |  |  |  |  |  | 4 | 7 | 6 |  |  |  |  |  |  |  |
| 3 | 2 |  | 6 |  |  |  |  |  |  |  |  |  |  |  |  |  |  |
| 8 |  | 2 |  |  |  |  |  |  |  |  |  |  |  |  |  |  |  |
| 13 |  |  | 1 |  |  |  |  | 2 |  |  | 6 |  |  |  |  |  |  |
| 14 |  |  | 3 | 4 |  |  |  |  |  |  |  |  |  |  |  |  |  |
| 19 |  |  |  | 2 |  |  |  |  |  |  |  |  |  |  |  |  |  |
| 29 |  |  |  |  | 2 |  |  |  |  |  |  |  |  |  |  |  |  |
| 33 |  |  |  |  | 2 |  |  |  |  |  |  |  |  |  |  |  |  |
| 34 |  |  |  |  | 2 |  |  |  |  |  |  |  |  |  |  |  |  |
| 35 |  |  |  |  |  |  | 7 |  |  |  |  |  |  |  |  |  |  |
| 36 |  |  |  |  |  | 2 |  |  |  |  |  |  |  |  |  |  |  |
| 37 |  |  |  |  |  |  |  |  |  |  |  |  |  | 2 |  |  |  |
| 43 |  |  |  |  |  |  |  |  |  |  |  | 3 |  |  |  |  |  |
| 46 |  |  |  |  |  |  |  |  |  |  |  |  | 3 |  |  |  |  |
| 47 |  |  |  |  |  |  |  |  |  |  |  |  | 4 |  |  |  |  |
| 48 |  |  |  |  |  |  |  |  |  |  |  |  |  |  | 7 |  |  |
| 50 |  |  |  |  |  |  |  |  |  |  |  |  |  |  |  | 4 | 6 |
