# Supplementary material for: Pleistocene glacial cycle effects on the phylogeography of the Chinese endemic bat species, Myotis davidii
Source: BMC Evol Biol. 2010 Jul 10;10:208. doi: 10.1186/1471-2148-10-208 (PMC3055248; doi:10.1186/1471-2148-10-208)
Supplement: Additional file 2 — AMOVA in mtDNA and microsatellite. The molecular variance analysis (AMOVA) in mtDNA and microsatellite of Myotis davidii were in three geographical regions. Regions chosen: Middle East Plain, Southwest Plateau, South Hills. Asterisks highlight hierarchical levels explain a significant proportion of the overall variance (P < 0.001). [file 1471-2148-10-208-S2.DOC]

**Additional file 2**

**AMOVA in mtDNA and microsatellite.** The molecular variance analysis (AMOVA) in mtDNA and microsatellite of *Myotis davidii* were in three geographical regions. Regions chosen: Middle East Plain, Southwest Plateau, South Hills. Asterisks highlight hierarchical levels explain a significant proportion of the overall variance (*P* < 0.001).

| Marker | Source of variation | Squares components of variation | Variance components | Percentage of variation | Fixation indices |
| --- | --- | --- | --- | --- | --- |
| mtDNA | Among clades | 736.593 | 10.880 | 64.82 | Φ_CT_ : 0.771* |
|  | Among populations/within clades | 235.302 | 2.061 | 12.28 | Φ_SC_ : 0.349* |
|  | Within populations | 353.683 | 3.844 | 22.90 | Φ_ST_ : 0.648* |
| nDNA | Among clades | 74.127 | 0.446 | 14.45 | Φ_CT_: 0.113* |
|  | Among populations/within clades | 134.544 | 0.511 | 16.57 | Φ_SC_: 0.124* |
|  | Within populations | 394.456 | 2.130 | 68.98 | Φ_ST_: 0.205* |
