# Supplementary material for: Pleistocene glacial cycle effects on the phylogeography of the Chinese endemic bat species, Myotis davidii
Source: BMC Evol Biol. 2010 Jul 10;10:208. doi: 10.1186/1471-2148-10-208 (PMC3055248; doi:10.1186/1471-2148-10-208)
Supplement: Additional file 3 — Estimates of genetic difference derived from both mtDNA and microsatellites. Above diagonal is Φst from mtDNA. Below diagonal is Fst from microsatellites. The significant values are marked with asterisks. The pairwise genetic difference within regions is in bold. Significance level = 0.05. [file 1471-2148-10-208-S3.DOC]

**Additional file 3**

**Estimates of genetic difference derived from both mtDNA and microsatellites.** Above diagonal is *Φ*_st_ of mtDNA. Below diagonal is *F*_st_ from microsatellites. The significant values are marked with asterisks. The pairwise genetic difference within regions is in bold. Significance level=0.05.

| Regions | Location | MEP | | | | | | SWP | | | | | | | | SH | | |
| --- | --- | --- | --- | --- | --- | --- | --- | --- | --- | --- | --- | --- | --- | --- | --- | --- | --- | --- |
|  |  | AH1 | AH2 | JS | ZJ | JX | CQ2 | CQ1 | GZ1 | GZ2 | HN | YN4 | YN3 | YN2 | YN1 | GD1 | GD1 | GX |
| MEP | AH1 | - | **0.159*** | **0.199*** | **0.007** | **0.171*** | **0.352*** | 0.769***** | 0.711***** | 0.748***** | 0.631***** | 0.697***** | 0.699***** | 0.752***** | 0.732***** | 0.760***** | 0.737***** | 0.685 |
|  | AH2 | **0.033*** | - | **0.237*** | **0.218*** | **0.293*** | **0.479*** | 0.872***** | 0.819***** | 0.849***** | 0.750***** | 0.833***** | 0.838***** | 0.868***** | 0.853***** | 0.855***** | 0.832***** | 0.784***** |
|  | JS | **0.015** | **0.045*** | - | **0.114*** | **0.081*** | **0.322*** | 0.686***** | 0.662***** | 0.677***** | 0.622***** | 0.623***** | 0.647***** | 0.680***** | 0.668***** | 0.686***** | 0.678***** | 0.651***** |
|  | ZJ | **0.007*** | **0.019*** | **0.008*** | - | **0.105*** | **0.361*** | 0.789***** | 0.743***** | 0.774***** | 0.683***** | 0.728***** | 0.744***** | 0.779***** | 0.763***** | 0.785***** | 0.768***** | 0.728***** |
|  | JX | **0.004*** | **0.021*** | **0.022** | **0.048** | - | **0.457*** | 0.808***** | 0.767***** | 0.793***** | 0.712***** | 0.763***** | 0.772***** | 0.802***** | 0.789***** | 0.802***** | 0.785***** | 0.749***** |
|  | CQ2 | **0.123*** | **0.173*** | **0.174*** | **0.229*** | **0.219*** | - | 0.899***** | 0.904***** | 0.959***** | 0.808***** | 0.964***** | 0.981***** | 0.988***** | 0.976***** | 0.979***** | 0.938***** | 0.871***** |
| SWP | CQ1 | 0.159* | 0.176***** | 0.101***** | 0.227***** | 0.196 | 0.268***** | - | **0.438*** | **0.727*** | **0.450*** | **0.975*** | **0.929*** | **0.981*** | **0.903*** | 0.974***** | 0.927***** | 0.849***** |
|  | GZ1 | 0.147* | 0.112***** | 0.099***** | 0.163***** | 0.151***** | 0.241***** | **0.098*** | - | **0.249*** | **0.261*** | **0.653*** | **0.627*** | **0.697*** | **0.639*** | 0.867***** | 0.831***** | 0.769***** |
|  | GZ2 | 0.149* | 0.103 | 0.122 | 0.161***** | 0.164***** | 0.273 | **0.220** | **0.095** | - | **0.454*** | **0.862*** | **0.849*** | **0.885*** | **0.830*** | 0.929***** | 0.900***** | 0.819***** |
|  | HN | 0.141* | 0.084***** | 0.074***** | 0.143***** | 0.130***** | 0.202***** | **0.065*** | **0.015** | **0.074** | - | **0.460*** | **0.417*** | **0.526*** | **0.483*** | 0.757***** | 0.723***** | 0.657***** |
|  | YN4 | 0.138* | 0.068 | 0.056 | 0.158***** | 0.101***** | 0.202***** | **0.144** | **0.050** | **0.168** | **0.026** | - | **0.878*** | **0.975*** | **0.681** | 0.967***** | 0.907***** | 0.812***** |
|  | YN3 | 0.105* | 0.079 | 0.069 | 0.120***** | 0.085***** | 0.256***** | **0.179*** | **0.062** | **0.158** | **0.062** | **0.056** | - | **0.122** | **0.132** | 0.963***** | 0.895***** | 0.789***** |
|  | YN2 | 0.120* | 0.108***** | 0.103***** | 0.114***** | 0.137***** | 0.264***** | **0.098*** | **0.023** | **0.109** | **0.022** | **0.015** | **0.036** | - | **0.142** | 0.972 | 0.920***** | 0.837***** |
|  | YN1 | 0.148* | 0.114***** | 0.130***** | 0.189***** | 0.176***** | 0.246***** | **0.106*** | **0.073** | **0.109** | **0.011** | **0.083** | **0.119** | **0.051** | - | 0.945***** | 0.897***** | 0.818***** |
| SH | GD1 | 0.253* | 0.155***** | 0.204***** | 0.239***** | 0.214***** | 0.209***** | 0.236***** | 0.146 | 0.168 | 0.099 | 0.173 | 0.168 | 0.132***** | 0.128 | - | **0.0012** | **0.378*** |
|  | GD2 | 0.275* | 0.175***** | 0.234***** | 0.270***** | 0.266***** | 0.211***** | 0.253***** | 0.200 | 0.172***** | 0.167***** | 0.239 | 0.236 | 0.217***** | 0.171***** | **0.024** | - | **0.347*** |
|  | GX | 0.276* | 0.237***** | 0.232***** | 0.271***** | 0.258***** | 0.255***** | 0.271***** | 0.196***** | 0.244***** | 0.193***** | 0.234***** | 0.240***** | 0.207***** | 0.165***** | **0.064** | **0.123*** | - |
